# Techniques of Primary Vaginoplasty in Young Adults with Differences of Sex Development and Female Identification

**DOI:** 10.3390/jcm11133688

**Published:** 2022-06-27

**Authors:** Verena Ellerkamp, Kristin Katharina Rall, Juergen Schaefer, Sara Brucker, Joerg Fuchs

**Affiliations:** 1 Department of Pediatric Surgery and Pediatric Urology, University Hospital Tübingen, D-72076 Tübingen, Germany; joerg.fuchs@med.uni-tuebingen.de; 2 Department of Gynecology and Obstetrics, University Hospital Tübingen, D-72076 Tübingen, Germany; katharina.rall@med.uni-tuebingen.de (K.K.R.); sara.brucker@med.uni-tuebingen.de (S.B.); 3 Department of Radiology, University Hospital Tübingen, D-72076 Tübingen, Germany; juergen.schaefer@med.uni-tuebingen.de

**Keywords:** disorders/differences of sex development, vaginoplasty, urogenital sinus, clitoroplasty, congenital adrenal hyperplasia, vaginal agenesis

## Abstract

**Background**: The ideal timing of genital surgery in differences/disorders of sex development (DSD) is controversial and differs according to the underlying type of DSD. Increasing numbers of persisting sinus as a result of delayed feminizing genitoplasty in DSD patients require interdisciplinary collaboration of pediatric surgeons/urologists and gynecologists. This study focusses on surgical techniques other than bowel vaginoplasties and results of gender assigning surgery in young adolescents. **Methods:** Data of adolescent and adult patients treated between 2015 and 2022 were analyzed retrospectively: underlying type of malformation, techniques of vaginoplasty, vaginal length and caliber, possibility of sexual intercourse, and temporary vaginal dilatation. **Results:** A total of 9 patients received a primary vaginoplasty at a median age of 16.75 years (range 10.3–29.25). The underlying anatomical conditions were persistent urogenital sinus (UGS) in 8 patients (3 patients with CAH, 2 patients with XY-DSD, 1 patient with cloacal malformation and missed UGS, 2 patients with UGS only). One patient had a MURCS association. Surgical techniques were total urogenital mobilization and perineal flap vaginoplasty in 4 patients, modified McIndoe vaginoplasty in 4 patients, and a laparoscopic vaginal pull-through in 1 patient. In a median follow-up of 45 months (2–84), all but 1 patient presented with physiological vaginal length and width. **Conclusions:** If possible, modern treatment concepts delay gender assigning surgery until the participation of the patient in the decision-making process is possible. Optimal treatment concepts are given by transfer of surgical techniques from pediatric urology/surgery by multidisciplinary teams. Techniques other than bowel vaginoplasties are favorable.

## 1. Introduction

The ideal timing of genital surgery in disorders/differences of sex development (DSD) is still controversial and differs according to the underlying type of DSD [1]. It is generally accepted that deferring surgery until a secure gender identity is achieved is a basic to obtaining a fundamental well-being of the individual.

The incidence of female genital malformation is low but differs according to the respective form. MRKH is the most common form with an incidence of 1:4000–1:5000 female live births [2]. Patients with this form of 46 XX-DSD are usually not quickly diagnosed after birth and grow up with a “normal” appearance of their female external genitals. Possible surgical techniques vary from primary vaginal dilatation to vaginoplasties (VP) (Vecchietti procedure, skin graft procedures, or bowel VP). There is no question of postponing surgery in these patients until adolescence or young adulthood. Similar, surgery in other cases of DSD presenting with urogenital sinus, such as mixed gonadal dysgenesis, must be postponed until the patients are certain about their gender identity and are able to make a decision about the corrective surgery.

More controversial is the optimal timing of surgery in the second most common DSD, congenital adrenal hypoplasia (CAH) with an incidence of 1:7500 female live births. Since CAH is classified in the group of DSD, the issue of age at genitoplasty has recently been discussed [3]. Some countries even try to prevent early surgery in these patients by law.

Accordingly, recommendations and evaluations for endocrinologic and psychological aspects of treatment in DSD patients are increasingly evidence-based [4]. Whereas recommendations for surgical therapy seem to remain largely vague.

Surgical principles depend on the individual anatomic findings: patients with low confluence urogenital sinus (UGS) are most widely operated with posterior skin flap vaginoplasty as introduced by Fortunoff et al. in 1964 [5]. In cases of high confluence UGS, the technique of total urogenital mobilization (TUM) introduced by Pena in 1997 markedly increased the cosmetic and functional outcome, this technique may be combined with a perineal flap or a longitudinally split sinus tissue as a mucosa lined introitus [6,7]. In patients with vaginal hypoplasia or agenesis the Vecchietti vaginoplasty, introduced in 1965, is the gold standard [8]; there is usually significant improvement after transfer to minimally invasive techniques and optimized instruments [9,10]. Alternatively, in these patients the neovagina may be created in the retrovesical space and lined with different types of tissue: the oldest techniques use a split thickness skin graft after blunt dissection of a retrovesical space and was introduced in 1937 according to Mc-Indoe-Reed [11]. The Davydov procedure creates the neovagina from an open or rather laparoscopic abdominal approach and uses peritoneal flaps as inner lining [12,13]. All of these techniques deal with the risk of obstructing cicatrization and may create the need for postoperative vaginal dilatation. Intestinal vaginoplasty with a sigmoid or ileal segment with its vascular pedicle offers an adequate vaginal length, a lower risk of shrinkage, and natural lubrification through mucosal secretion [14,15,16]. Alongside all of these theoretical advantages, intestinal vaginoplasty is a complex procedure with a higher morbidity due to resection loss of intestinal length and anastomosis of bowel with complication rates up to >30% [14]. Main disadvantages may be excessive vaginal mucus discharge, unpleasant smell due to persisting gut biome, diversion colitis, and malignancy risk.

Despite improving surgical techniques with good cosmetic results after surgery in childhood many reports deal with relevant quotes of patients’ dissatisfaction of functional outcome in their adult live [17]. Most authors meanwhile recommend genital surgery in infancy only in cases with low confluence sinus in 46, XX DSD-girls but to postpone surgery in all other cases to puberty or later [18]. Nevertheless, studies of genital surgery beyond infancy and childhood are mainly concerned with bowel VP in transgender patients or deal with techniques of redo-VP [19,20,21,22].

In the current study we want to share our experience in primary VP-techniques in female identified adolescents with DSD and also introduce small modifications of well-known techniques.

## 2. Materials and Methods

**Figure 1:**
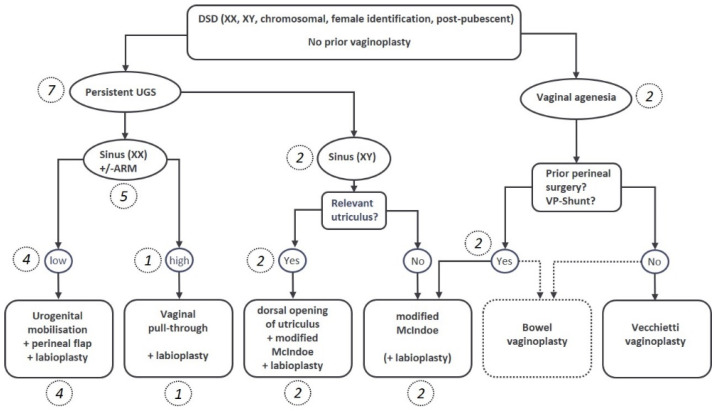
Flowchart for decision making regarding the individual favored surgical method. The respective number of patients in brackets and italics.

Patient charts for all female patients treated by the interdisciplinary DSD center of excellence between 2015 to 2022 were retrospectively analyzed. Patients were preoperatively evaluated as described elsewhere [23]. In short, genitoscopy with catheter placement in the urethra, vagina/utriculus under anesthesia and genitography and/or pelvic MRI with contrast media were performed for diagnostic reasons. Treatment concepts were then determined in our interdisciplinary DSD board consisting of pediatric surgeons, gynecologists, endocrinologists, and psychologists. According to our Tübinger DSD flowchart (Figure 1), the respective favored VP-technique was discussed with the adult patients, or the teenage patients and their families. VP was only indicated in cases of clear gender identification, the ability to consent, and - in CAH patients—earliest from the onset of pubarche.

In cases of low urogenital sinus (UGS) in CAH or XY-DSD patients with a sufficient vagina or utriculus, a perineal flap VP was preferred. Surgery was performed in lithotomy position. The urethra and vagina were intubated with catheters in Seldinger technique cystoscopically. In cases of clitoromegaly, the glans clitoris was circumcised, and a nerve-preserving resection of the corpora cavernosa was realized as described elsewhere [24]. The UGS was longitudinally opened to the division in urethra and vagina/utriculus. The distal urethra and vagina were then mobilized, the spatium rectovaginale was controlled with rectal Hegar’s dilatators to avoid any injury to the rectum or vagina. Based on the first description by Fortunoff in 1964 [5], a perineal reversed U-flap with plenty of fat tissue was lifted and the dorsal wall of the vagina was incised until the vagina could be intubated with a Hegar’s dilatator 18–20. The perineal flap was then inserted in the dorsal V-shaped vaginal defect with resorbable sutures, and the urethra was closely inserted to the reduced clitoris. Skin closure in the form of a labioplasty was performed.

In cases with a high UGS and adequate vaginal tissue, a laparoscopic mobilization of the uterus and vagina with closure of the urethrovaginal confluence/fistula was performed as described previously [25]. In short, in the same setting, a labioplasty was performed, the sinus was used as urethra and the reconstructed vagina was pulled-through and inserted into the reconstructed introitus.

In cases with insufficient utriculus or complete absence of the paramesonephric ducts modified McIndoe VP was favored, Vecchietti VP was not recommended because of former perineal surgery (anorectal malformation, ARM) or for other reasons. The preparation of the vaginal cave was performed as described in the original description of the technique in 1938 [11]. Briefly, a cross-shaped incision in the vaginal groove of the vulva was performed and the vaginal cavity is formed by blunt dissection. Different to the original McIndoe VP, we did not place any skin grafts.

As a novelty, in cases of small utriculus/vagina and short common channel of the UGS, perineal flap technique and modified McIndoe technique were combined: the UGS was prepared as for perineal flap VP as described above. The short and tight utriculus was then incised in its dorsal wall in a longitudinal fashion and an additional blunt dissection for widening and lengthening was performed before the perineal flap was inserted.

In all cases, vaginal measurements were determined at the end of surgery and individual vaginal dummies were ordered as previously described (VagiTom, Gomaringen, Germany) [23]. The neovagina was packed with compresses soaked in estrogen for 5 days, the urinary catheter was left in place for 5 to 7 days, pain management included epidural catheters for 5 days combined with NSAR. Cefalosporines were administered for 7 days. After 5 days, the vaginal dummies were placed and the patients were instructed to wear these stents for the first months continuously, reducing the wearing time stepwise afterwards, until complete epithelialization was achieved after about 6 months. Sexual intercourse is possible after 4–6 weeks.

All of the procedures were performed by a stable team of a pediatric surgeon and a gynecologist.

For outcome evaluation, the underlying malformation, ages and prior surgery of the pelvis, surgical techniques, measurements of the urogenital sinus, and vaginal length and width after genital repair, complications, as well as duration of vaginal dilatations were reviewed. For descriptive statistics, SPSS 26 was used. The study was approved by the Ethical committee of the University of Tuebingen, Germany (No 263/2020BO2).

## 3. Results

A total of 9 patients were referred within the time period with a median age of 16.75 years (range 10.3–29.25), anatomic conditions and underlying diseases are listed in Table 1. All but one patient had not been in our service since childhood or diagnosis. Seven patients had a 46, XX karyotype and two patients had a 46, XY karyotype (each one SF-1 defect, steroid 5-alpha reductase type 2 deficiency).

**Figure 2:**
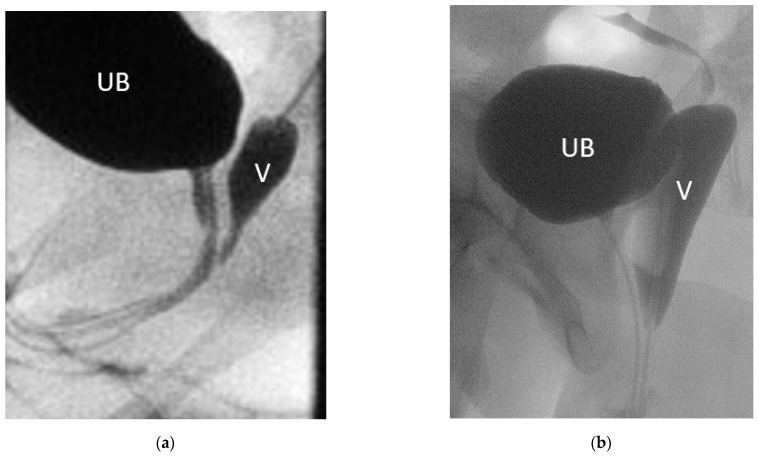
Genitogram of the same patient with (**a**) 2 months and (**b**) 14 years. UB = urinary bladder; V = vagina.

**Figure 3:**
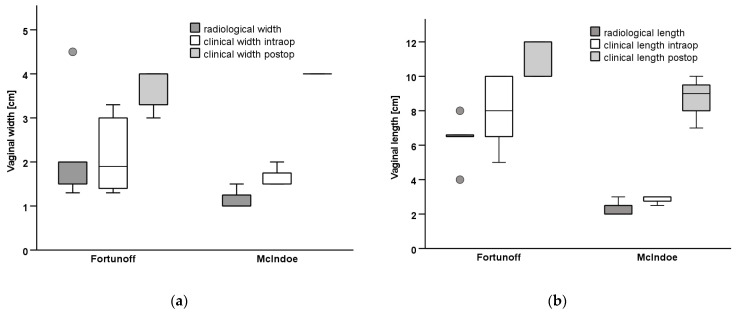
Measurements of the vagina radiological (MRI/MCU) vs. intraoperative before VP and in follow-up postoperatively. (**a**) Vaginal width, (**b**) Vaginal length.

Six patients had prior surgery, two of each had correction of ARM, clitoroplasty, and gonadectomy in their infancy or childhood (Table 1). Moreover, 8 patients had a persistent urogenital sinus, 6 of them with a short common channel of a median length of 2.5 cm (range 1–4); 2 patients had a long common channel (5, and 7 cm), 1 had only a rudimentary vagina and hypoplastic uterus. In one patient with CAH, the comparison of genitography in the early infancy with that after menarche shows how menstruation bleeding can stretch the vagina (Figure 2). In comparison, the lengths and widths of the vagina/utriculus measured radiologically in MRI and MCU are slightly smaller in most patients than in the intraoperative digital measurement during the final correction (Figure 3).

**Figure 4:**
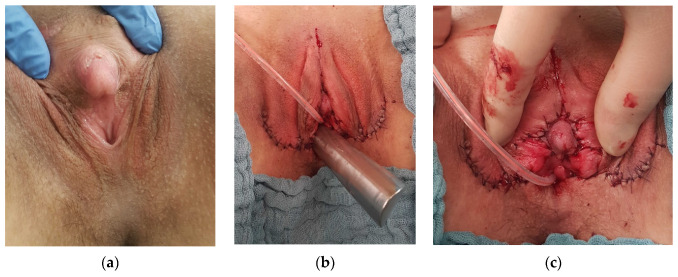
(**a**) Pre- and (**b**,**c**) postoperative aspect of perineal flap vaginoscopy and clitoroplasty.

In seven patients, vaginal tissue (5 patients), or utriculus, respectively (2 patients), were sufficient for VP. In 4 patients with a low sinus (3 CAH), partial urogenital mobilization with perineal flap VP and labioplasty were performed (Figure 4).

**Figure 5:**
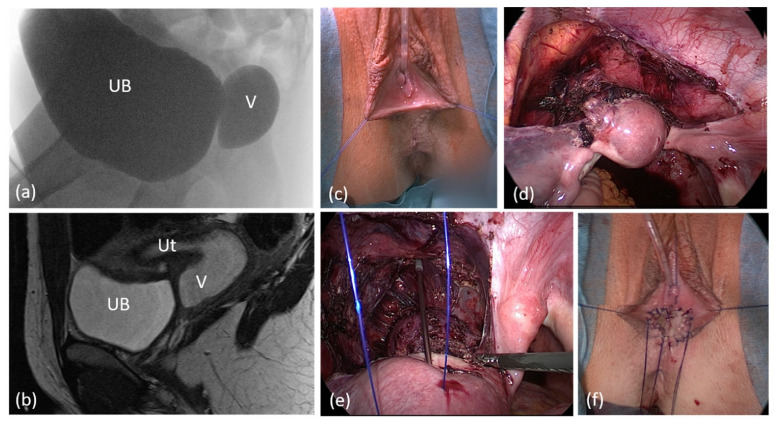
Laparoscopic pull-through in a case of unrecognized cloacal malformation with high urogenital sinus. (**a**) Genitogram (**b**) sagittal pelvic MRI. (**c**) Appearance of Introitus preoperatively. (**d**), (**e**) Laparoscopic view after mobilization of the uterus and proximal vagina. (**f**) Introitus after the vaginal pull-through; the vagina was calibrated with hegar 18, length 9 cm. UB = urinary bladder; V = vagina; Ut = uterus.

One laparoscopic vaginal pull-through procedure with closure of a fistula between the proximal urethra and vagina was performed in a patient with unrecognized cloacal malformation with undetected high urogenital sinus. Due to low ARM with vestibular fistula, a PSARP procedure had been realized in childhood. This patient had suffered from severe dysmenorrhea and recurrent urinary tract infections (UTI) over years until a hematometra and missing introitus was diagnosed (Figure 5).

**Figure 6:**
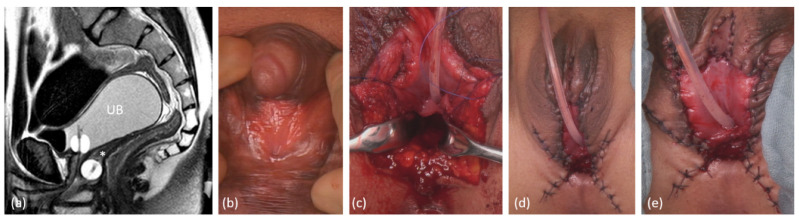
Genitoplasty in a patient with 46,XY DSD (steroid 5-alpha reductase type 2 deficiency) with the use of the utriculus as ventral neovaginal wall. (**a**) preoperative sagittal MRI, UB = urinary bladder; * = utriculus, both splinted with a foley catheter, short common channel. (**b**) preoperative aspect. (**c**) Opening of the sinus, dorsal longitudinal opening of the utriculus and extension of the neovagina with a Fortunoff flap (**d**), (**e**) Postoperative aspect after additional nerve-sparing reduction in the clitoral cavernous corpora.

In two cases with XY-DSD and persistent sinus/male urethra with hypospadia and utricle, the latter was used for reconstruction of the neovagina (Figure 6).

**Figure 7:**
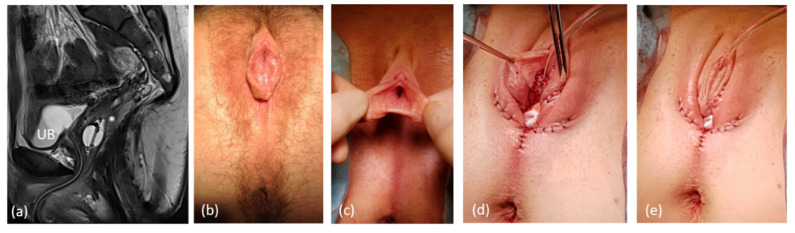
Genitoplasty in a patient with former correction of ARM via abdomino-peritoneal rectoplasty and late diagnosis of abnormally absent paramesonephric (Müllerian) ducts. (**a**) Sagittal MRI UB = urinary bladder; * = vaginal relict, both splinted with a foley catheter, long common channel. (**b**,**c**) preoperative clinical aspect. (**d**,**e**) postoperative appearance.

Two patients with no sufficient vaginal tissue/utriculus were not suitable for Vecchietti vaginoplasties due to the following circumstances: one adult patient with a history of ARM with correction in early childhood through abdomino-sacroperineal pull-through presented with symptoms of primary painless amenorrhea and sinus-like outer genital appearance. In combination with a rudimental uterus, she was diagnosed as MRKH with rudimental urogenital sinus. A high and extremely small vagina was insufficient for vaginal reconstruction. We performed a labioplasty/introitusplasty with the sinus walls in combination with a McIndoe neovagina without skin transplantation (Figure 7). Due to the rigidity of the perineal tissue the depth of the dissection for the vaginal cavity was limited to 6 cm. In another patient with MURCS, the Vecchietti technique was not considered because of a ventriculo-peritoneal shunt. In this patient, a McIndoe neovagina without skin transplantation was also realized.

The median follow-up was 45 months (range 1–84). All patients performed vaginal dilatations postoperatively over a period of 6–12 months with custom-made vaginal phantoms in ascending sizes. All of the patients have normal total vaginal width of 3.5 to 4 cm, and normal lengths with a median of 10 cm (range 7–12) (Figure 3). Concerning the ability for cohabitation, 5 patients have not yet attempted to do (2 felt too young, 3 have no partner), but all of them had vaginal dimensions that would allow sexual intercourse. Four patients have unproblematic intercourse, one of them feels some length limitation. No complication occurred in any of the patients.

## 4. Discussion

With the ongoing discussion about the ideal age of gender affirming surgery in patients with DSD, psychologic aspects and the ideal interdisciplinary treatment in terms of hormonal substitution are the main focus. In addition to good psychological and endocrinological care, late operations are intended to prevent gender dysphoria. Although it seems perfectly logical to postpone genital surgery to an age when gender identity has developed, there is no evidence of what extent development and life satisfaction are affected by growing up with ambiguous genitalia. Interestingly, surveys of adolescent and adult DSD-patients concerning their preferences on the ideal age of genital surgery were inconsistent. Preferences considerably varied by diagnostic category, gender, history of surgery, and contact with support groups [26]. Especially in CAH women, in retrospect many favor early gender affirming surgery and even basically question the classification of CAH in the group of DSD [3,26,27,28]. However, this issue is not only discussed on a medical socio-cultural level but even entered legislation in some countries [3]. Following other countries, in Germany, for example, legislation is being drafted to prohibit early surgical genital corrections in all cases of DSD, including patients with CAH. In order to be able to consent, a minimum age of 14 years is required. For earlier genital reconstruction an interdisciplinary DSD-board voting together with a family court decision are needed before any genital correction in all patients with DSD [29].

Regardless of these ethical aspects, medical aspects continue to be discussed in terms of the ideal age for genital correction, unless there are compelling health considerations that influence the decision. Surgical techniques depend on the anatomical conditions such as the length of the urogenital sinus and the available vaginal tissue or persistent utriculus [5,7,30,31]. When postponing surgery especially in CAH-patients, some surgeons recommend realizing corrective surgery before menarche to avoid hematometra, and endometriosis, related to obstructed menstruation [3]. However, obstructing menstruation is generally rather rare, we saw it in one patient with undetected high sinus and low ARM. However, many of these patients will normally undergo menstrual drainage without complication via the UGS [32,33]. On the contrary, hematocolpos as complication in CAH-patients is much more often seen after failed VP [34]. Moreover, patients with high UGS even may profit from some obstruction and pubertal hormone increase with an enlargement of vaginal size and some descent of the confluence as shown by others and our own herein reported patient [35].

Other authors vote for even earlier genital surgery in girls with Prader stage 3 or greater at the age of 6 to 12 months [3,36,37]. One argument is an easier availability and better quality of tissues during the first months of life: the maternal estrogen circulating in this early period may lead to a supposedly lower risk of obstructing scars [30,38]. On the other hand, studies showed that estrogenic effects on the genitals of newborns dissipate as early as by the fourth postnatal week, resulting in a loss of vulvar skin thickness and maturation index [39,40]. These findings are contrary to the theory that genital operations within the first 6–12 months of life may still benefit from maternal estrogen. Furthermore, in most studies the median age at surgery in CAH patients was >18 months, depicting that very many surgeons do not even operate on the female genitals during the period of supposed maternal estrogenization [41]. Interestingly, one argument made for early genitoplasty in CAH girls is the lack of a cohort of late primary CAH repair [38]. This still is true but, at least we hereby provide courses of three adolescents with genito-/vaginoplasties after menarche. The tissue quality in fact did not play a negative role during surgery; neither did the postoperative healing, nor the outcome. In another study, a 2-staged repair in 41 CAH girls with clitoroplasty in infancy but vaginoplasty during puberty described a better size of perineum and vagina with resulting easier creation of the posterior vaginal arch, an excellent functional outcome and low rate of necessary redo-VP of 12% [42]. In studies with VP in infancy, vaginal stenoses are indicated with 27% [43]. As there is no evidence showing if early or late VP is superior, the information of the parents of a newborn with CAH about possible operating times and the associated advantages and disadvantages should be discussed open to results considering the respective legal situation. In addition, psychological studies will help to clarify possible disadvantages for children identified as female, to grow up with an enlarged clitoris and without a vagina to reach puberty age. At the same time, these had to be compared with the psychological stress of repeated genital surgery in childhood.

If the vagina or utriculus is of small size and not available for TUM and perineal flap, techniques such as McIndoe-VP enable creation of a neovagina without the disadvantages and risks of intestinal VP. This technique also may be considered in patients with vaginal agenesis after perineal/pelvine surgery such as former anorectoplasty or with ventriculo-peritoneal shunt as a contraindication for Vecchietti-VP. Many modifications of the McIndoe-technique have been introduced and led to a further increase in the rate of satisfactory outcome from 80–100% [44]. Instead of split-thickness skin grafts, several other tissues have been described, such as human amnion, subcutaneous abdominal flaps, labial skin flaps, and autologous in vitro cultured vaginal tissue [44,45]. The huge experience with Vecchietti vaginoplasties prompted us to dispense with a skin-graft, resulting in good overall epithelialization within the follow-up after six weeks [46]. Our experience is confirmed by a study of Marzieh et al. of successful McIndoe vaginoplasty without skin grafts in 25 patients [47]. In cases of existing utriculus or small vagina and low confluence we performed the skin-graft-free McIndoe-VP but used the existing small vagina/utriculus as starting point of the vaginal cave and performed the blunt dissection after a longitudinal dorsal incision of the utriculus.

With the described techniques, normal vaginal lengths and width resulted in all patients with respect to informative overviews of normal vaginal dimensions of healthy women [48,49]. The functional outcome is satisfying, but a limitation is that in the short follow-up period, the patients did not all attempt to engage in sexual intercourse.

In our small series, one woman complained about the vaginal length with some limitation in sexual intercourse after McIndoe-VP without skin graft, however, she has painless intercourse.

However, recent studies have shown that vaginal size is not the only determinant of sexual satisfaction. Women with normal vaginas may also have significant cohabitation difficulties, which may be due to both scarring and pain and may have psychic causes after frequent corrective surgeries [50,51]. The functional outcome in means of sexual satisfaction and ability to cohabitation has much more impact than vaginal measurements. In the short follow-up period, the patients of our cohort did not all have a partner or were quite young; therefore, this aspect could only be evaluated to a limited extent.

## 5. Conclusions

VP after menarche in CAH patients is feasible; there is no technical need of performing gender assigning surgery in early infancy in these patients. Accordingly, the families concerned should be supported and advised to keep an open mind about the possibility of surgical corrections in adolescence. In cases of absent vagina or utriculus, skin-graft-free McIndoe-VP is a straightforward option. It may be also used as a simple enlargement of a very small vagina or utriculus.

## Figures and Tables

**Table 1:** Overview of the patients. CAH = congenital adrenal hyperplasia; CH = hypertrophic clitoris Cl = cloacae; UGS = urogenital sinus; PF = perineal flap; CP = clitoroplasty; UV fistula = urethrovaginal fistula; RV = rectovaginal fistula, VP = vaginoplasty; CRS = caudal regression syndrome; PSARP = posterior sagittal Anorectoplasty, SF1 = SF-1 defect; 5αRD2 = 5-Alpha-reductase 2 deficiency; Mod McIndoe = Modification of McIndoe vaginoplasty.

No	Caryotype	Anatomic Condition	Underlying Condition	Prior Surgery	VP Techniques
1	46, XX	UGS, CH	CAH	-	PUM + PF, CP
2	46, XX	UGS	CAH	CP	PUM + PF
3	46, XX	UGS	CAH	CP	PUM + PF
4	46, XX	UGS	ARM/cloaca	PSARP	Laparoscopic assisted vaginal pull-through
5	46, XX	UGS + absent paramesonephric ducts	ARM/cloaca	Abdominoperineal anorectoplasty	Mod McIndoe
6	46, XX	UGS		-	PUM + PF
7	46, XX	Vaginal agenesis	MURCS	VP-Shunt	Mod McIndoe
8	46, XY	UGS, CH	SF1	gonadectomy	PUM + Mod McIndoe, CP
9	46, XY	UGS, CH	5αRD2	gonadectomy	PUM + Mod McIndoe, CP

## Data Availability

Not applicable.
